# On the Olfactory Powers of the Turkey Buzzard

**Published:** 1846-06

**Authors:** N. Mendenhall

**Affiliations:** North Carolina


*On the Olfactory Powers of the Turkey Buzzard.* By N. Mendenhall, M. D., of North Carolina. (In a letter to Prof. Dunglison.)

In reference to the sense by which the Turkey Buzzard is guided in the search of its food, the experiments of Audubon, &c., would appear to be conclusive, inasmuch as they seem to prove that this bird is not guided by the sense of smell, but that it is led by the sense of sight only. I am not aware of the precise circumstances under which these experiments were conducted, yet from facts which are known to myself, I cannot but infer that there must be some fallacy in the conclusions to which they lead.

It is a very common practice in our part of the Union, when any animal dies, to drag it into some deep ravine in the neighbourhood, and there let it putrify and be devoured by the buzzards. These ravines or hollows are generally surrounded by bushes and trees of a very luxuriant growth, and notwithstanding the foliage is so thick that the rays of the sun cannot penetrate it,—thus rendering it in the highest degree improbable that any thing under them could be distinguished by a bird in the air—yet we find the dead animal surrounded by buzzards—so far as I know, never before, but as soon as it begins to emit a perceptible odour.

I could relate instances, too, of snakes and other small animals being killed and thrown out into a thicket of bushes and weeds so dense as to render any small object invisible at the distance of two or three feet; yet whenever the smell of putridity became marked, these vultures were attracted to it in considerable numbers. What makes me the more certain that they were led here by the sense of smell only is the fact, that when they arrived within a short distance of the dead animal, they would alight and look about, and, not finding it, make shorter and shorter gyrations, until seemingly led on by a stronger and stronger scent, they reached the spot where they could gratify also their *sight* and *taste*.

The arguments given above, however, are not absolutely conclusive, for it may be said we are not *positively* certain, that the buzzard was not here directed by the sense of sight, though this is in a very high degree improbable; and I will therefore give another instance on the authority of my room mate, (E. B. Clark,) which, it seems to me, added to the circumstances before mentioned, and others of a similar kind, ought to put this matter at rest: A number of swine, and among them a brood of little pigs, were in the habit of lying under an old barn. The barn had in a good measure lost its roof, but the floor of the part under which the swine slept was complete, and, moreover, piled up with boards, &c., so as to render it impossible that any thing could be seen from above; and it was raised only about eighteen inches from the ground. One of the pigs died in this situation. Some short time after the workmen were engaged in hauling hay by this barn, and seeing a good many buzzards perched on a fence near it, which returned after being frightened away, and on coming still nearer, perceiving the smell themselves, they were led to inquire into the source of it. After considerable search the dead pig was found far under the floor—so far, that in this instance there can be no doubt, that the buzzards were attracted towards it by some other sense than that of vision.

Nor do I think that the observation of the English professor (Owen) alluded to by you, is of small weight in determining this question, not saying, by the way, that the question itself is of any great importance. I believe it is a principle very generally conceded, that the amount of sensibility in any part is in direct proportion to the amount of nervous matter distributed to that part, at least when the nerves are in a state of health. When, then, we consider the beautiful uniformity which prevails in the works of creation, and at the same time recollect that the Almighty architect formeth nothing in vain, we cannot but conclude, that whatever the observation of Audubon, &c., may have shown in respect of the *vision* of the bird alluded to, its sense of *smell* is in a high degree acute, and that frequently it is guided by this alone.

With great respect,
N. Mendenhall.

[In relation to this subject we may cite the following remarks of the distinguished naturalist, Mr. Charles Darwin, M. A., F. R. S., in his "Journal of Researches into the Natural History and Geology of the countries visited during the voyage of H. M. S. Beagle round the world," which has been recently printed in this country, New York, 1846.]

"When an animal is killed in the country, [Chile,] it is well known, that the condors, like other carrion-vultures, soon gain intelligence of it, and congregate in an inexplicable manner. In most cases it must not be overlooked that the birds have discovered their prey, and have picked the skeleton clean before the flesh is in the least degree tainted. Remembering the experiments of M. Audubon on the little smelling powers of carrion-hawks, I tried in the above-mentioned garden the following experiment. The condors were tied, each by a rope, in a long row at the bottom of a wall; and having folded up a piece of meat in white paper, I walked backwards and forwards, carrying it in my hand at the distance of about three yards from them, but no notice whatever was taken. I then threw it on the ground, within one yard of an old male bird; he looked at it for a moment with attention, but then regarded it no more. With a stick I pushed it closer and closer, until at last he touched it with his beak: the paper was then instantly torn off with fury, and at the same moment every bird in the long row began struggling and flapping its wings. Under the same circumstances, it would have been quite impossible to have deceived a dog. The evidence in favour of and against the acute smelling powers of carrion vultures is singularly balanced. Professor Owen has demonstrated that the olfactory nerves of the turkey-buzzard (Cathartes aura, are highly developed, and on the evening when Mr. Owen's paper was read at the Zoological Society, it was mentioned by a gentlemen that he had seen the carrion-hawks in the West Indies on two occasions, collect on the roof of a house where a corpse had become offensive from not having been buried: in this case the intelligence could hardly have been acquired by sight. On the other hand, besides the experiments of Audubon and that one by myself, Mr. Bachman has tried in the United States many varied plans, showing that neither the turkey-buzzard (the species dissected by Professor Owen) nor the gallinazo find their food by smell. He covered portions of highly offensive offal with a thin canvass cloth, and strewed pieces of meat on it; these the carrion-vultures ate up, and then remained quietly standing, with their beaks within the eighth of an inch of the putrid mass, without discovering it. A small rent was made in the canvass, and the offal was immediately discovered; the canvass was replaced by a fresh piece, and meat again put on it, and was again devoured by the vultures without their discovering the hidden mass on which they were trampling. These facts are attested by the signatures of six gentlemen, besides that of Mr. Bachman.*"

"*Loudon's Magazine of Natural History, vol. vii."

